# International registry collaboration and statistical approaches

**DOI:** 10.1080/17453674.2018.1487210

**Published:** 2018-07-23

**Authors:** Ove Furnes

**Affiliations:** The Norwegian Arthroplasty Register, Department of Orthopaedic Surgery, Haukeland University Hospital, Bergen and Department of Clinical Medicine (K1), Faculty of Medicine, University of Bergen, Norway

International registry collaboration is often hampered by regulations preventing transferal of individual patient data between countries. In this issue of Acta, Paxton et al. (2018) report the use of meta-analysis in registry research and compare it with results based on individual-patient level. The meta-analysis approach is well known in medical scientific work, but is not well known among orthopedic surgeons (Arends et al. 2008). Using a meta-analysis approach each registry conducts analysis on its own data given a pre-specified protocol and data syntax. The risk estimates are combined in a meta-analysis. The approach was first used in international hip and knee replacement registry research in the United States. The Food and Drug Administration (FDA) funded collaboration between 6 national and regional registries of the International Consortium of Orthopaedic Registries (Sedrakyan et al. 2014, Cafri et al. 2015). Several studies were published from this collaboration reporting results on articulation and fixation of hip prostheses and stabilization of knee replacements (Sedrakyan et al. 2014). An important question is whether this approach leads to the same results and estimates as use of individual patient-level data, which have been used in individual registry studies and the Nordic Arthroplasty Register Associations studies (Havelin et al. 2009, Robertsson et al. 2010, Johanson et al. 2017). In the study by Paxton the meta-analysis approach and individual-level data gave the same results, and adding on one additional registry to the study gave more precise estimates.

The meta-analysis approach demands that a detailed protocol is prepared and a statistical syntax is made by the leading analysis center; this syntax must be used by each individual registry participating in the study. This approach is not flexible and new sub-analysis and small corrections to the protocol demand that the syntax must be redone centrally and new analysis performed. Different statistical approaches can be used such as fixed-effects and random-effects models (Arends et al. 2008, Cafri et al. 2015). Individual-level analysis is more flexible and preferred, but is often impossible since not all registries are allowed to share individual-level data even if they are anonymized. Thus, the US registry was not allowed to contribute to the analysis with individual-level data for privacy and security reasons in the Paxton study. However, the meta-analysis approach as demonstrated by Paxton gave the same results as the individual-level analysis. This is reassuring, as it may convince more registries to contribute data to multinational studies. With the meta-analysis approach each registry has control of its own data and data-ownership issues are of less concern. The known problems of confounding of unknown variables in observational studies such as confounding by indication cannot be accounted for in either individual-level studies or meta-analysis. Both study approaches use time-to-event analysis approaches such as Kaplan–Meier and Cox analysis.

Ove Furnes

*The Norwegian Arthroplasty Register, Department of Orthopaedic Surgery, Haukeland University Hospital, Bergen and Department of Clinical Medicine (K1), Faculty of Medicine, University of Bergen, Norway*

ove.furnes@helse-bergen.no
